# Neuronal self-injury mediated by IL-1β and MMP-9 in a cerebral palsy model of severe neonatal encephalopathy induced by immune activation plus hypoxia-ischemia

**DOI:** 10.1186/s12974-015-0330-8

**Published:** 2015-05-30

**Authors:** Alexandre Savard, Marie-Elsa Brochu, Mathilde Chevin, Clémence Guiraut, Djordje Grbic, Guillaume Sébire

**Affiliations:** Laboratoire de Neurologie Pédiatrique, Université de Sherbrooke, Sherbrooke, QC Canada; McGill University, 2300 Tupper street, H3H 1P3 Montreal, QC Canada

**Keywords:** Apoptosis, Cerebral palsy, Chemokines, Hypoxia-ischemia, Interleukin-1, Matrix metalloproteinase-9, Necroptosis, Neonatal encephalopathy

## Abstract

**Background:**

Inflammation due to remote pathogen exposure combined to hypoxia/ischemia (HI) is one of the most common causes of neonatal encephalopathy affecting at-term or near-term human newborn, which will consequently develop cerebral palsy. Within term-equivalent rat brains exposed to systemic lipopolysaccharide (LPS) plus HI, it was previously showed that neurons produce IL-1β earlier than do glial cells, and that blocking IL-1 was neuroprotective. To further define the mechanisms whereby IL-1 exerts its neurotoxic effect, we hypothesize that IL-1β plays a pivotal role in a direct and/or indirect mechanistic loop of neuronal self-injury through matrix metalloproteinase (MMP)-9.

**Methods:**

An established preclinical rat model of LPS+HI-induced neonatal encephalopathy was used. In situ hybridization, ELISA, and immunolabeling techniques were employed. Selective blocking compounds allowed addressing the respective roles of IL-1 and MMP-9.

**Results:**

In LPS+HI-exposed forebrains, neuronal IL-1β was first detected in infarcted neocortical and striatal areas and later in glial cells of the adjacent white matter. Neuronal IL-1β played a key role: (*i*) in the early post-HI exacerbation of neuroinflammation and (*ii*) in generating both core and penumbral infarcted cerebral areas. Systemically administered IL-1 receptor antagonist (IL-1Ra) reached the brain and bound to the neocortical and deep gray neuronal membranes. Then, IL-1Ra down-regulated IL-1β mRNA and MMP-9 neuronal synthesis. Immediately post-HI, neuronal IL-1β up-regulated cytokine-induced neutrophil chemoattractant (CINC-1), monocyte chemoattractant protein-1 (MCP-1), and inducible nitric oxide synthase. MMP-9 would disrupt the blood–brain barrier, which, combined to CINC-1 up-regulation, would play a role in polymorphonuclear cell (PMN) infiltration into the LPS+HI-exposed brain. IL-1β blockade prevented PMN infiltration and oriented the phenotype of macrophagic/microglial cells towards anti-inflammatory and neurotrophic M2 profile. IL-1β increased the expression of activated caspase-3 and of receptor-interacting-protein (RIP)-3 within infarcted forebrain area. Such apoptotic and necroptotic pathway activations were prevented by IL-1Ra, as well as ensuing cerebral palsy-like brain damage and motor impairment.

**Conclusions:**

This work uncovered a new paradigm of neuronal self-injury orchestrated by neuronal synthesis of IL-1β and MMP-9. In addition, it reinforced the translational neuroprotective potential of IL-1 blockers to alleviate human perinatal brain injuries.

## Background

Hypoxia-ischemia (HI) combined to inflammation is an important pathophysiological component of neonatal encephalopathy (NE) affecting up to 1 % of newborns. Indeed, combined pathogen-induced inflammation complicated by HI was recognized as one of the most common causes of human perinatal brain damage [1–8]. Moderate and severe forms of NE lead either to death or lifelong neurodisabilities such as cerebral palsy (CP) or other neurobehavioral disabilities including learning difficulties [1, 4]. Combination of inflammation/infection plus HI has been reproduced experimentally in rodents at neurodevelopmental stages equivalent to early and late preterm development of human neonates—i.e., in rat pups between P1 and P7 [5, 9–13]. Inflammation/infection and HI have also recently been modeled in rats at a neurodevelopmental stage (P12) corresponding to full-term human neonate development. Profound differences in the neuropathological impacts from lipopolysaccharide (LPS) plus HI-induced patterns of innate immune responses have been shown between P1 and P12 rats [13, 14]. Importantly, these neuropathological differences reproduce those observed between preterm and term human newborns [14]. Thus, LPS+HI-induced brain injury of P12 rat pups is a relevant model for the study of term human NE [15].

We previously showed that, within term-matched rat brains exposed to systemic LPS+HI, neurons produce IL-1β earlier than do glial cells, and that IL-1 receptor antagonist (IL-1Ra) was neuroprotective [15]. To further define the neurotoxic mechanisms of IL-1 in the immature brain, we hypothesize that IL-1 induces directly and/or indirectly neuronal self-injury involving matrix metalloproteinase (MMP)-9, blood–brain barrier (BBB) disruption, and polymorphonuclear cell (PMN) infiltration.

## Materials and methods

### Rat model

Our pre-clinical model was designed as previously described [15]. Briefly, Lewis dams were obtained from Charles River Laboratories (Saint-Constant, QC, Canada) between gestational day 14 (G14) and 16 (G16). P12 pups received a single intraperitoneal (ip) injection of LPS (200 μg/kg diluted in 50 μL of saline) from *Escherichia coli* or saline. Ischemia was induced 4 h after LPS administration by permanent ligature of the right common carotid artery followed by 8 % oxygen exposure at 37+/− 1 °C for 1.5 h. A control (Ctl) group underwent no surgery, and a sham surgery group was submitted to the common carotid artery exposure without ligature. Because we found no observable difference in the results of these two groups, both were combined and identified as the Ctl group. Pups from each litter were randomized in three different experimental groups (Ctl, LPS+HI, LPS+HI+IL-1Ra) independently of sex and weight. The end of hypoxia is referred to as 0 h. Pups were sacrificed at 4 h (P12), 24 h (P13), 48 h (P14), and 8 days (P20) post-HI. Each session of surgery was performed in groups of 10–20 animals from 2–4 litters; our data of mortality was the mean ± SEM of mortality from each of these groups (total of 78 pups in LPS+HI condition; 72 pups in LPS+HI+IL-1Ra condition; 38 pups in Ctl condition). Some rats were kept alive until P120 for motor behavior. Rats were euthanized by rapid decapitation. The ethical review board of the Sherbrooke University approved this experimental design (protocol #147-11R). The care of animals followed internationally recognized guidelines.

### Blocking experiments using IL-1Ra and MMP-9 inhibitor (SB3CT)

IL-1 receptor antagonist (IL-1Ra) was used at a concentration of 200 mg/kg. This dose has been demonstrated as the most effective in terms of neuroprotection in adult stroke models as well as in the above-mentioned model [15, 16]. The first injection was given ip, 30 min before LPS injection. Five other injections were given every 12 h thereafter. SB3CT (Sigma, ON, Canada) was used at a concentration of 12.5 mg/kg. This dose was proven effective in a mouse model of middle cerebral artery occlusion. The first injection was given before surgery, a second 4 h after HI, and another every 12 h thereafter for a total of six injections.

### Histology

Brains were fixed in paraformaldehyde (PFA) 4 % at room temperature, paraffin-embedded, and cut in 5-μm slices using a microtome for histological studies. Hematoxylin-eosin (H&E) staining was used to visualize and quantify brain injuries. ImageJ analysis software (National Institutes of Health (NIH) Image, http://rsbweb.nih.gov/nih-image/) was used to measure the surface of the right hemisphere on coronal sections located at the epicenter of the infarct (Bregma −1.00). The right hemisphere surfaces of LPS- and/or HI-exposed rats were then compared with those of Ctl. So-called core vs penumbra areas of brain infarcts were defined as previously described [15]. Briefly, core injuries were associated with infarcted areas bearing cavitary lesions, whereas penumbra lesions were identified as regions surrounding the core where pycnotic neurons or loss of normal neuronal architecture were observed.

### Immunohistochemistry (IHC) and immunofluorescence (IF)

IHC and IF were performed as previously described [5, 15]. Briefly, sections were incubated overnight at 4 °C with the primary antibodies. The antibodies used are detailed in Table 1. The appropriate HRP-conjugated or Alexa Fluor-conjugated secondary Abs were used for each primary Ab and incubated for 1 h at room temperature. Labeling was revealed using diaminobenzidine (DAB) (Roche, QC, Canada). Slides were counterstained with hematoxylin. IF slides were mounted using a DAPI-containing medium (Invitrogen, ON, Canada). Negative controls consisted of an additional set of sections, treated similarly but without the primary antibody. Labeled cells were counted, and cytokine expression was analyzed using the ImageJ analysis software (NIH Image, http://rsbweb.nih.gov/nih-image/). The percentage of cells meant the number of a given cell type on the total number of cells per field (magnification ×40).

**Table 1:** List and features of antibodies

Antibody	Company—reference number	Dilution
Anti-DIG-AP	Roche—11093274910	1:3000
Anti-Albumin	MP Biomedicals—55727	1:100
Anti-NeuN	Millipore—MAB377	1:100
Anti-Iba-1	Abcam—ab15690	1:200
Anti-PMN	Cedarlane—CLAD51140	1:200
Anti-iNOS	SCBT—sc-49058	1:100
Anti-MCP-1	Chemicon—AB1834P	1:100
Anti-CINC-1	Abcam—ab10365	1:100
Anti-claudin-5	Abcam—ab53765	1:100
Anti-RIP-3	SBCT—sc-135170	1:30
Anti-caspase-3	Millipore—AB3623	1:20
Anti-IL-1β	Serotec—AAR15G	1:250
Anti-MMP-9	Chemicon—AB19016	1:100
Anti-rabbit-HRP	Serotec—STAR54	1:100
Anti-goat-HRP	SCBT—sc-2350	1:100
Anti-rabbit-Alexa Fluor conjugated	Invitrogen—A11012	1:500
Anti-mouse-Alexa Fluor conjugated	Invitrogen—A11005	1:500
Anti-chicken-Alexa Fluor conjugated	Invitrogen—A11039	1:500

### ELISA

Protein extracts were prepared from right hemisphere forebrains as previously described [15]. ELISAs were performed on these protein extracts using ELISA Kits (R&D System, MN, USA), as previously described [14].

### In situ hybridization

mRNA encoding for IL-1β was detected and localized on brain sections using dioxigenin-UTP (DIG) labeled riboprobes. The IL-1β DNA template was amplified using primers with specific restriction enzymes: IL-1β BamHI forward 5′- AGT CCT GGA TCC ATG GCA ACT GTC CCT GAA CT -3′ and IL-1β EcoRI reverse 5′- GGC CGC GAA TTC AGC TCA TGG AGA ATA CCA CT -3′. DIG-labeled single-stranded RNA was transcribed from commercially available plasmid vectors according to manufacturer instructions (Roche, Laval, QC, Canada, 1175025). The in situ hybridization protocol used was that of Dr Jasna Kriz [17]. Briefly, slides were dried, post-fixed in 4 % paraformaldehyde, and digested by proteinase K, after which brain sections were washed in water, in a solution of 0.1 M triethanolamine (TEA, pH 8.0), and then acetylated in 0.25 % acetic anhydride in 0.1 M TEA. Hybridization of brain sections with the riboprobe was performed overnight at 72 °C. Slides were washed in standard saline citrate (1XSSC: 0.15 M NaCl, 15 mM trisodium citrate buffer, pH 7.0) and were then incubated with anti-DIG-AP antibodies overnight at 4 °C. Next, slides were incubated with NBT/BCIP solution, for color development; at room temperature for 16 h. Slides were then washed with TBST and mounted with DAKO Cytomation Glycergel Mounting Medium. Staining intensities of IL-1β mRNA were studied by colorimetric analysis performed with ImageJ software as previously described [15].

### Behavioral tests

Circling behavior test was performed using the open field test apparatus. Rat pups at P14 were placed in the center of the open field and videotaped for 1.5 min. The movements of the pups were scored as following: 0 = absence of circling, 1 = partial circling, 2 = total circling [15]. The open field test was used to determine spontaneous locomotor activity and exploratory behavior from P100 to P120, as described previously [5]. Elevated body swing test was used, as previously described, to determine long-term motor impairment from P100 to P120 [18]. The movements of rats suspended by their tails were video-recorded. We set the threshold at an angle of 90° for an efficient upswing. Latency before the first efficient upswing, the side of the efficient upswing, and the total number of efficient upswings were recorded and compared across experimental conditions. The Turning in Alley test was performed at P100–P120, as previously described, to assess any potential persisting shift of lateralization due to motor impairment [19]. Rats were placed facing the end of a closed alley: the duration and the side of the turning were recorded.

### Data analysis

Data are presented as means ± SEM. Results across experimental conditions were compared using the unpaired *t* test with Welch correction. The statistical significance level was set at *p* ≤ 0.05. Data from the Ctl group and sham group were combined, as well as male and female data, because they are not significantly different.

## Results

### Distribution of IL-1Ra in LPS+HI-exposed brains

**Fig. 1:**
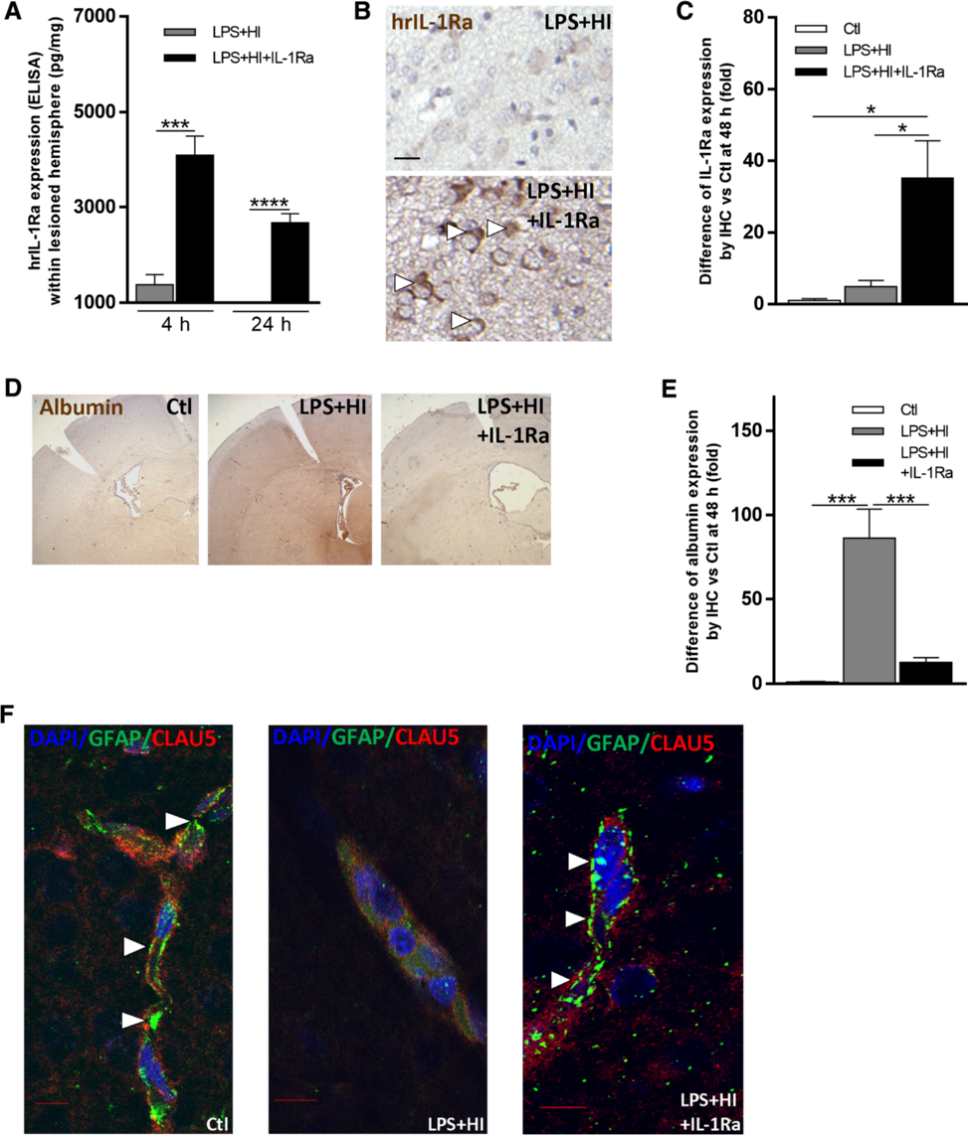
Impact of IL-1Ra on the BBB after LPS+HI exposure. Titer (ELISA) of hrIL-1Ra (pg/mg) in the right cerebral hemisphere at 4 and 24 h after LPS+HI ± IL-1Ra exposure (**a**). Immunohistochemistry (IHC) staining of hrIL-1Ra associated with neurons (*arrowhead*) in LPS+HI ± IL-1Ra-exposed brains at 48 h (**b**). Relative hrIL-1Ra expression (IHC) at 48 h in LPS+HI ± IL-1Ra-exposed frontoparietal cortical areas compared to control (**c**). IL-1Ra prevented albumin (IHC staining) entry within the brain at 48 h (**d**). IL-1Ra decreased IHC relative expression of albumin in the right cerebral hemispheres 48 h after LPS+HI exposure (**e**). IL-1Ra prevented LPS+HI-induced BBB disruption (loss of linear co-localization of GFAP and claudin-5 (*arrowheads*)) as seen by confocal microscopy following immunofluorescent (IF) staining of claudin-5 and GFAP of blood vessels of right frontoparietal middle neocortical blood vessels of LPS+HI ± IL-1Ra-exposed rats at 4 h post-HI (**f**). IF images are representative of the blood vessels in damaged areas. *Error bars* represent means ± SEM, *scale bar* = 10 μm, three to six animals per conditions, unpaired *t* test with Welch correction, * *p* ≤ 0.05, *** *p* ≤ 0.001, **** *p* ≤ 0.0001. Abbreviations: *GFAP* glial fibrillary acidic protein, *Clau5* claudin-5

hrIL-1Ra (ip injected) crossed the blood–brain barrier (BBB), bound to neurons (Fig. 1a–c), and rapidly reached a high intracerebral titer (above 2500 pg/mg) at 4 h and 24 h post HI in brains exposed to LPS+HI+IL-1Ra condition (Fig. 1a). To test whether the BBB might be permeated by an LPS+HI insult, as well as its preventability by IL-1Ra, we looked for an albumin leakage through the forebrain BBB at 4 h and 48 h post-HI ± IL-1Ra. A functional disruption of BBB in LPS+HI condition and its prevention by IL-1Ra was demonstrated by a significantly higher albumin infiltration in LPS+HI than in LPS+HI+IL-1Ra exposed brains (Fig. 1d, e). A structural disruption of BBB was also shown in LPS+HI-exposed brains at 4 h after post-LPS+HI (Fig. 1f): LPS+HI exposure disrupted the tight junction protein claudin-5 and GFAP linear/continuous expression of BBB. IL-1Ra prevented the disruptive effect of LPS+HI on the BBB (Fig. 1f).

**Fig. 2:**
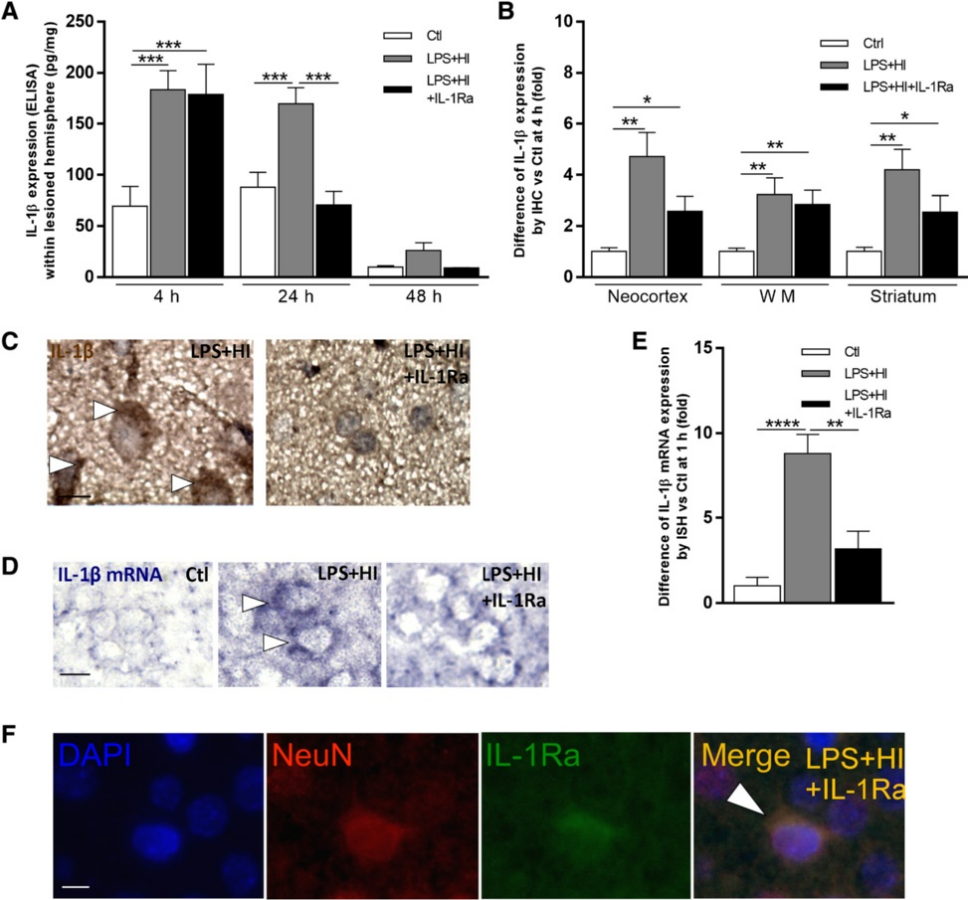
Effect of IL-1Ra on IL-1β in LPS+HI-exposed brains. IL-1Ra decreased titers (ELISA) of IL-1β (pg/mg) at 24 and 48 h following LPS+HI exposures (**a**). IL-1Ra decreased IL-1β relative IHC expressions in the right frontoparietal neocortex, white matter (WM), and striatum at 4 h after LPS+HI exposures (**b**). IL-1Ra prevented IHC staining of IL-1β in the neocortex of LPS+HI-exposed pups at 4 h post-HI (**c**). IL-1Ra prevented neuronal IL-1β mRNA expression (*arrowhead*) in the neocortex 1 h after LPS+HI as detected by in situ hybridization (**d**). IL-1Ra decreased relative expression of IL-1β mRNA in right frontoparietal cortical neurons 1 h post LPS+HI (**e**). IF double staining of NeuN+IL-1Ra+ neurons at 4 h post-HI in LPS+HI+IL-1Ra condition (**f**). *Error bars* represent means ± SEM, *scale bar* = 10 μm, four to six animals per conditions, unpaired *t* test with Welch correction, * *p* ≤ 0.05, ** *p* ≤ 0.01, *** *p* ≤ 0.001. Abbreviations: *WM* white matter

To test whether IL-1Ra immunoreactivity detected at the surface of neurons might interfere with IL-1 function, we compared the levels of auto-activation of IL-1β synthesis between LPS+HI vs LPS+HI+IL-1Ra conditions (Fig. 2a–f). Compared to LPS+HI alone, forebrains exposed to LPS+HI+IL-1Ra presented a two to threefold drop in IL-1β mRNA (in situ hybridization) at 1 h post-HI (Fig. 2e) and in IL-1β expression (ELISA) at 24 h and 48 h post-HI (Fig. 2a). This IL-1β synthesis and its drop under IL-1Ra treatment was from neuronal cells as shown by in situ hybridization at 1 h post-HI (Fig. 2d, e) and by IHC in both core and penumbra at 4 h post-HI (Fig. 2c). IL-1Ra binding to neurons was shown by IF (NeuN+IL-1Ra+) double labeling at 4 h post-HI (Fig. 2f). The discrepancy between the diminished IL-1β detection on neuronal cell bodies by IHC at 4 h (Fig. 2c) and its absence of decrease at the same time by ELISA (Fig. 2a) might be due to the rapid binding of IL-1Ra on the neuronal IL-1Rs that would limit other IL-1 ligands binding at 4 h but without any decrease in the amount of free extracellular IL-1 at this early—but not later—time points (Fig. 2a, c).

### IL-1Ra protects against LPS+HI-induced brain injury and ensuing neurobehavioral morbidity and mortality

**Fig. 3:**
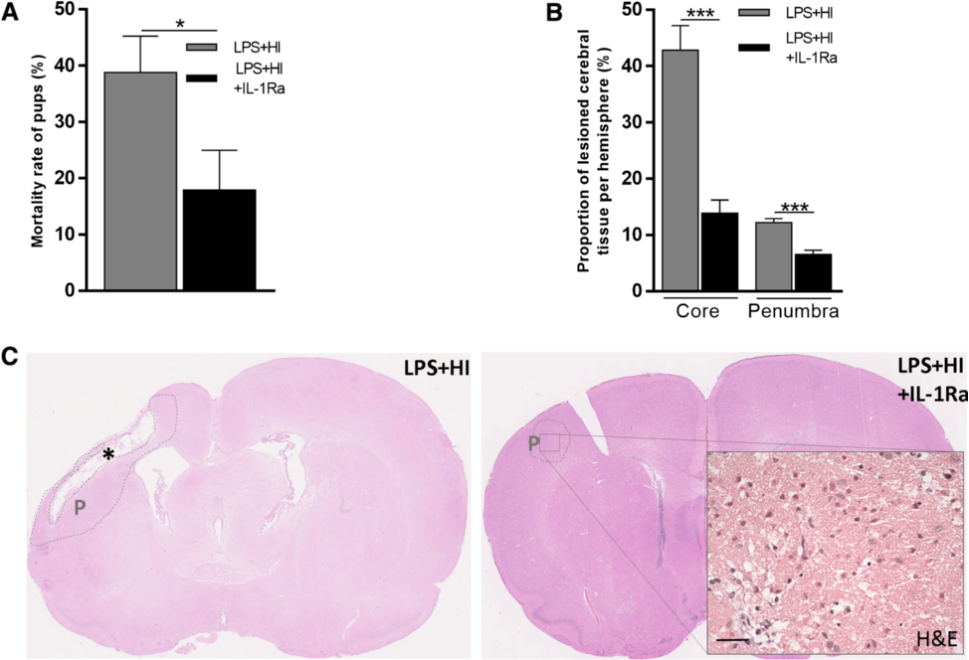
Effect of IL-1Ra on neuronal morbidity and mortality of LPS+HI exposed pups. IL-1Ra decreased the percentage of mortality of pups (all the mortality of rats occurred during hypoxia, between 1 and 1 h 30 min of hypoxia) (**a**) and the right hemisphere percentage of tissue loss in forebrains exposed to LPS+HI (**b**). Macroscopic view of brain damage at P20 by H&E stained on coronal sections of pup exposed to LPS+HI ± IL-1Ra (**c**). Core lesions are indicated by *asterisks*, and penumbra lesions are indicated by *P*. These lesions were reduced by IL-1Ra treatment. *Error bars* represent means ± SEM, *scale bar* = 50 μm, six to eight animals per condition, unpaired *t* test with Welch correction, * *p* ≤ 0.05, *** *p* ≤ 0.001. Abbreviations: *H&E* hematoxylin and eosin

**Fig. 4:**
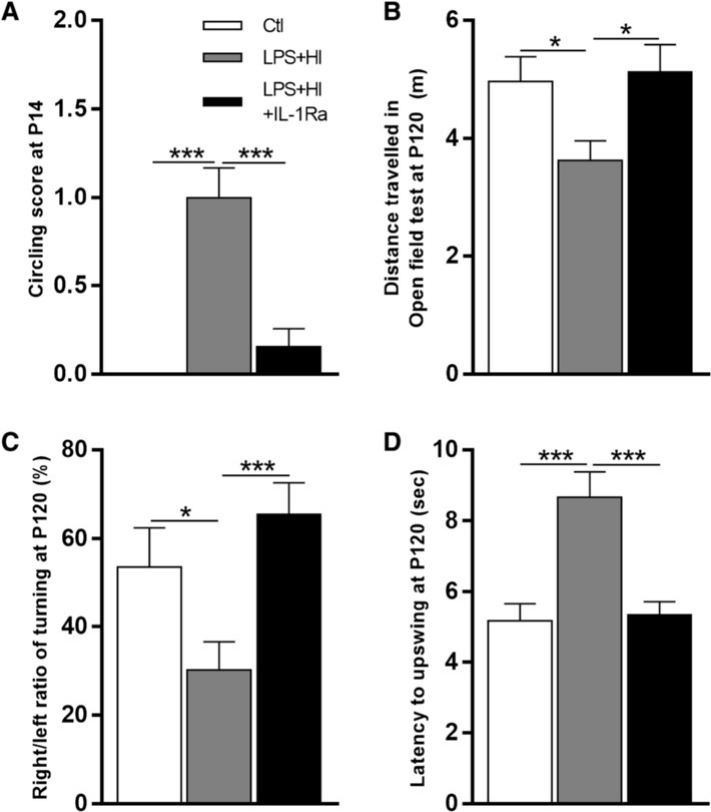
Effect of IL-1Ra on motor behavior of LPS+HI-exposed rats at P14 and P120. IL-1Ra decreased the circling score of animals 48 h after the exposition to LPS+HI (**a**). At P120, IL-1Ra decreased the distance traveled in the open field (**b**), right/left ratio in Turning in Alley (**c**), and latency to upswing (**d**) in elevated body swing tests of rats exposed to LPS+HI. *Error bars* represent means ± SEM, ten to twenty animals per conditions, unpaired *t* test with Welch correction, * *p* ≤ 0.05, *** *p* ≤ 0.001. Abbreviations: *m* meter, *sec* second

**Fig. 5:**
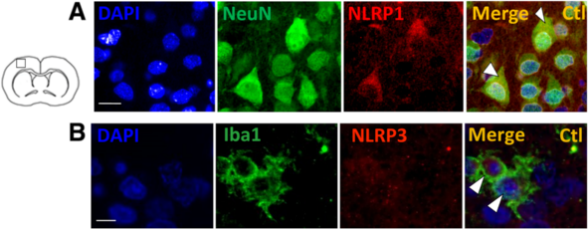
Expressions of NLRP1 and NLRP3 in neocortical neurons. IF staining of co-localizing NeuN+NLRP1+ neurons (*arrowheads*) in the neocortex at P12 in control rats (**a**). IF staining of co-localizing Iba1+NLRP3+ macrophage/microglia (*arrowheads*) in the neocortex at P40 in control rats (**b**). *Scale bar* = 10 μm, three to four animals per conditions, unpaired *t* test with Welch correction, ** *p* ≤ 0.01, **** *p* ≤ 0.0001. Abbreviations: *NLRP* nod-like receptor protein, *NeuN* neuronal nuclei, *Iba-1* ionized calcium-binding adapter molecule 1

IL-1Ra reduced LPS+HI-induced mortality (Fig. 3a). The comparison of H&E staining between brains exposed to LPS+HI vs LPS+HI+IL-1Ra showed that IL-1Ra prevented the extent of brain injury (Fig. 3b, c). IL-1Ra exerted a neuroprotective effect on both the core and penumbra components of ischemic gray and WM injuries of the forebrain (Fig. 3b, c). Behavioral experiments showed that LPS+HI-induced motor impairment was prevented by IL-1Ra at both early juvenile (P14) and late adult (P120) time points (Fig. 4). This curbing of brain injury, and subsequent motor behavior impairment, was associated with an early drop of neuronal IL-1β mRNA expression within the infarcted area, from 1 h post-LPS+HI (Fig. 2d, e). This intra-cerebral drop in IL-1β mRNA and IL-1β synthesis under IL-1Ra treatment might result from decreased activation of either neuronal nuclear factors *e.g.* NF-κB, or nod-like receptors (NLRs) of the inflammasome, or both. It has previously been shown that the inflammasome is activated during adult brain injury and that NLRP1 and NLRP3 (NLRs) are detected in adult neurons and macrophage/microglia, respectively [20]. Our results showed that NLRP1 and NLRP3 remained undetectable at P1, but that NLRP1 start to be expressed in cortical and subcortical neurons at P12 (Fig. 5a); 50 % of NLRP1+ cells we observed at P12 were also NeuN+; thus, glial cells at this P12 stage of development in rats also expressed NLRP1. NLRP3 was not detected at P1 or P12, but was at P40 in macrophage/microglial cells though not in neurons (Fig. 5b). This means that NLRP1 driven inflammasome might play a role within LPS+HI-exposed neurons to trigger IL-1β release.

### IL-1Ra interferes with key neuroinflammatory mediators and prevents LPS+HI-induced PMN recruitment and amoeboid M1 pro-inflammatory polarization of macrophage/microglia

**Fig. 6:**
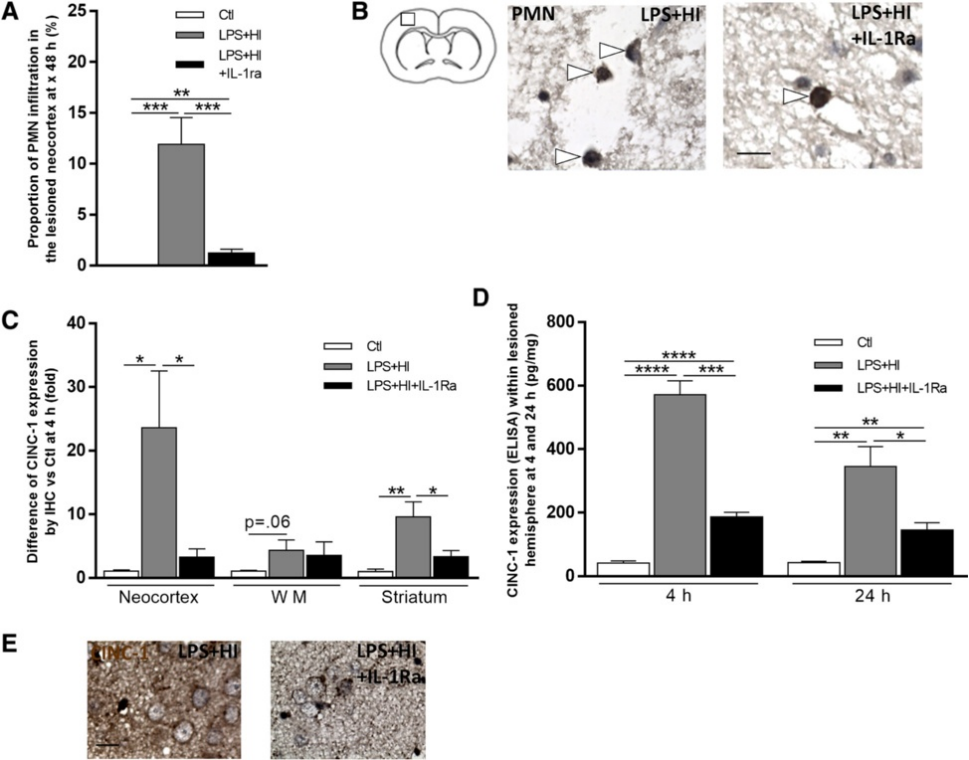
Effects of IL-1Ra on CINC-1 chemokine and PMN infiltration in LPS+HI-exposed brains. Decreased total cell percentage of PMN infiltration with IL-1Ra in the right frontoparietal neocortex at 48 h after LPS+HI (**a**). IL-1Ra prevented PMN (*arrowhead*) infiltration (IHC staining) in the infarcted neocortex of LPS+HI-exposed rats (**b**). Decreased relative expression of CINC-1 in the brain of LPS+HI+IL-1Ra-exposed rats compared to LPS+HI at 4 h (**c**). IL-1Ra decreased the titer (ELISA) of CINC-1 (pg/mg) within the right cerebral hemisphere at 4 and 24 h after LPS+HI (**d**). IHC of CINC-1 in the right frontoparietal neocortex 4 h after LPS+HI ± IL-1Ra (**e**). *Error bars* represent means ± SEM, *scale bar* = 10 μm, four to six animals per conditions, unpaired *t* test with Welch correction, * *p* ≤ 0.05, ** *p* ≤ 0.01, *** *p* ≤ 0.001, **** *p* ≤ 0.0001. Abbreviations: *PMN* polymorphonuclear neutrophils, *CINC-1* cytokine-induced neutrophil chemoattractant 1; *WM* white matter

IL-1Ra prevented PMN infiltration within LPS+HI-exposed brains at 48 h (Fig. 6a, b). IL-1Ra also curbed the induction of IL-1β-driven PMN-recruiting chemokine CINC-1 (cytokine-induced neutrophil chemoattractant-1) at 4 and 24 h—by respectively threefold and twofold—within the LPS+HI injured forebrain (Fig. 6c, d).

**Fig. 7:**
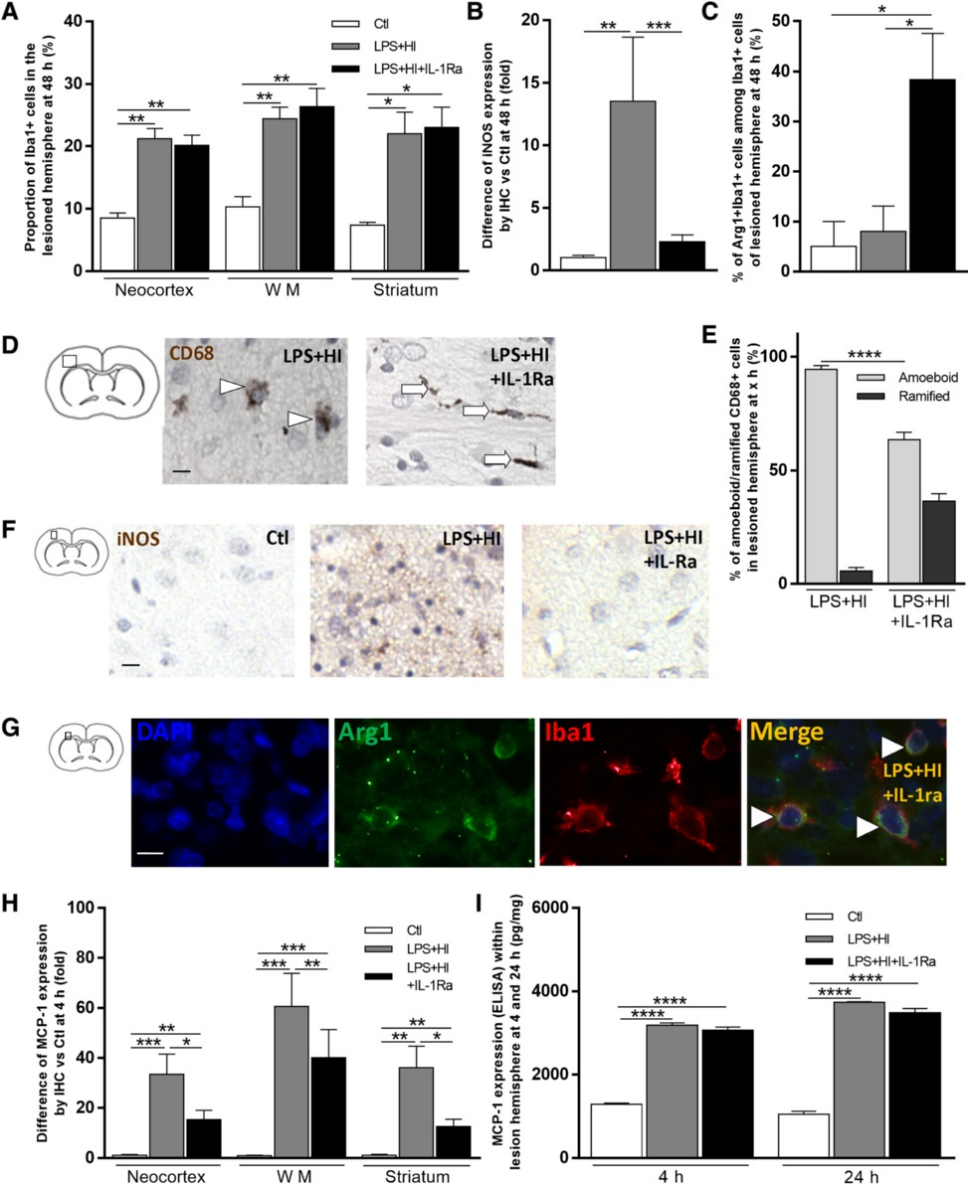
Effects of IL-1Ra on MCP-1 chemokine, M1/M2 microglia/macrophages infiltration, and polarization in LPS+HI-exposed brains. Total cell percentages of Iba1+ cells in LPS+HI ± IL-1Ra-exposed frontoparietal cortex, white matter, and caudate putamen nuclei of the right hemisphere at 48 h (**a**). Decreased relative expression of iNOS (IHC staining) in the right frontoparietal neocortex of LPS+HI+IL-1Ra-exposed rats at 48 h compared to LPS+HI (**b**). Increased Iba1+ cell percentage of Iba1+Arg1+ cells in the frontoparietal WM of the right hemisphere of LPS+HI+IL-1Ra-exposed rats at 48 h (**c**). IL-1Ra prevented macrophage activation by keeping them in a ramified morphology (*arrows*), instead of amoeboid (activated) morphology (*arrowheads*), as shown by CD68 staining (IHC) at 48 h after LPS+HI (**d**). Change in the CD68+ cell percentage of activated/quiescent macrophages in LPS+HI ± IL-1Ra-exposed brains (**e**). IL-1Ra prevented IHC staining of iNOS at 48 h after LPS+HI (**f**). IL-1Ra increased IF staining of Iba1+Arg1+ microglia/macrophages (*arrowhead*) in the frontoparietal white matter of the right hemisphere at 48 h (**g**). IL-1Ra decreased relative expression of MCP-1 (IHC staining) in the brain of LPS+HI-exposed rats at 4 h (**h**). MCP-1 titer (pg/mg) measured by ELISA within the right cerebral hemisphere at 4 and 24 h after LPS+HI (**i**). *Error bars* represent means ± SEM, *scale bar* = 10 μm, four to six animals per conditions, unpaired *t* test with Welch correction, * *p* ≤ 0.05, ** *p* ≤ 0.01, *** *p* ≤ 0.001, **** *p* ≤ 0.0001. Abbreviations: *Iba1* ionized calcium-binding adapter molecule 1, *Arg1* arginase 1, *iNOS* inducible nitric oxide synthase, *MCP-1* monocyte chemotactic protein 1, *CD68* cluster of differentiation 68, *WM* white matter

IL-1Ra had no effect on the total number of macrophage detected within LPS+HI injured brain areas (Fig. 7a). The proportion of inducible nitric oxide synthase (iNOS) positive cells significantly decreased within LPS+HI+IL-1Ra—compared to LPS+HI—affected forebrains (Fig. 7b, f). On the other hand, following IL-1Ra administration, the proportion of arginase 1 (Arg1)-positive microglia/macrophages—i.e., M2 polarized macrophages—significantly rose within LPS+HI+IL-1Ra- compared to LPS+HI-exposed forebrains (Fig. 7c, g). Under IL-1Ra, brain macrophage/microglia bore a quiescent ramified morphology compared to an activated amoeboid morphology in the absence of IL-1Ra (Fig. 7d, e). Thus, following LPS+HI aggression, IL-1Ra shifted from M1 towards M2 phenotype, i.e., towards non-inflammatory and possibly neuroprotective macrophagic/microglial polarization [21]. The induction of an IL-1β-driven macrophage chemokine synthesis, namely MCP-1, was also assessed. A significant rise of MCP-1 was observed following LPS+HI exposure (Fig. 7h, i). IL-1Ra decreased the proportion of cells expressing MCP-1 in neocortical, WM, and striatal areas at 4 h post-HI (Fig. 7h) but the global quantification of MCP-1 expressed within the lesioned cerebral hemisphere did not differ between LPS+HI vs LPS+HI+L-1Ra conditions (Fig. 7i). This is likely due to unbalanced intra- vs extra-cellular distribution of MCP-1 depending on IL-1Ra concentration with an overall lack of impact of IL-1Ra on macrophagic/microglial density (Fig. 7a) in cortical and subcortical affected areas (Fig. 7a).

### Role of neuronal MMP-9 in LPS+HI-induced neurotoxicity

**Fig. 8:**
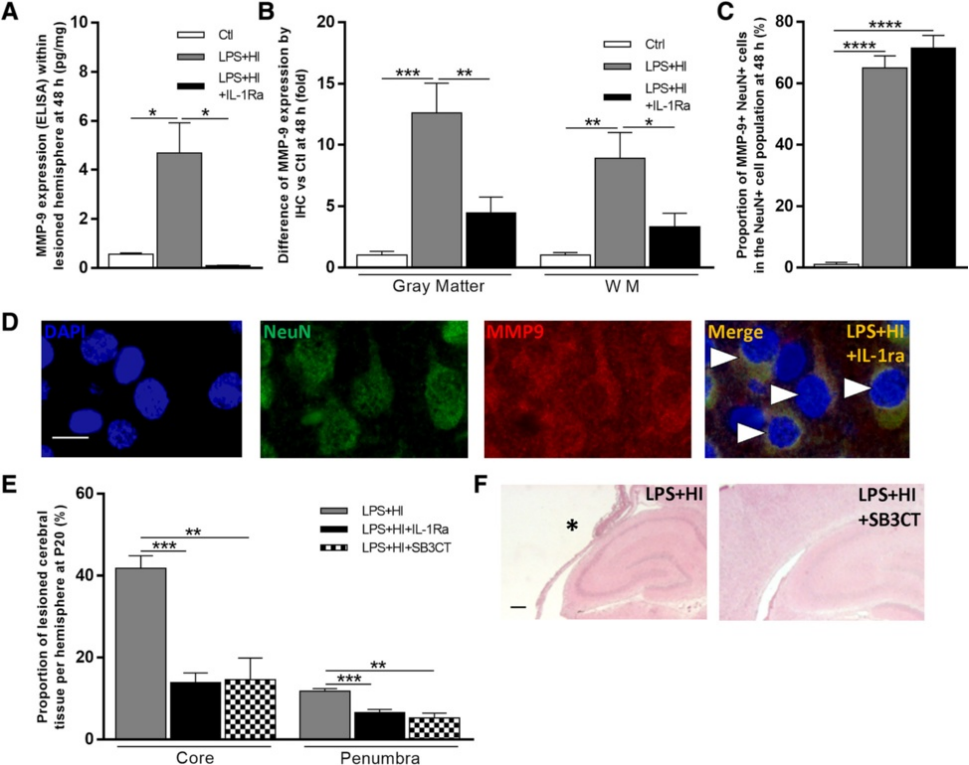
Effect of IL-1Ra on MMP-9 expression in LPS+HI-exposed brains. IL-1Ra decreased the titer of total MMP-9 (ng/mg) measured by ELISA within the right cerebral hemisphere at 48 h after exposure to LPS+HI (**a**). This decrease was confirmed by immunostaining of total MMP-9 expression at 48 h in LPS+HI ± IL-1Ra-exposed brains in *gray* and *white matters* (**b**). The proportion of double positive NeuN+MMP-9+ among NeuN+ neurons (**d**) from the frontoparietal neocortex of the right hemisphere at 48 h after LPS+HI was not affected by IL-1Ra exposures (**c**). Decreased right hemisphere percentage of tissue loss in LPS+HI-exposed forebrains treated by IL-1Ra or by SB-3CT at P20 (**e**). SB3CT prevented the extent of brain lesions after LPS+HI exposure in the same measure as IL-1Ra (**f**). Core of the lesion was indicated by *asterisks. Error bars* represent means ± SEM, *scale bar* = 10 μm, four to six animals per conditions, unpaired *t* test with Welch correction, * *p* ≤ 0.05, ** *p* ≤ 0.01, *** *p* ≤ 0.001, **** *p* ≤ 0.0001. Abbreviations: *MMP9* matrix metallo-protease 9, *NeuN* neuronal nuclei, *WM* white matter

Among IL-1β-induced effectors of BBB disruption and neural cell death, MMP-9 has been shown to be particularly important [22]. Forebrains submitted to LPS+HI showed a rise in MMP-9 production compared to Ctl at 48 h post-HI (Fig. 8a, b). Sixty percent of cortical neurons were MMP-9 positive compared to a mere 1 % under Ctl conditions (Fig. 8c). All MMP-9 positive cells were NeuN positive (Fig. 8d) in cortical and deep gray neurons, whereas no double labeling was observed between MMP-9 and astroglial (GFAP) or macrophage/microglial (Iba1) cell markers (data not shown). MMP-9 expression within the LPS+HI exposed brain was fully prevented by IL-1Ra treatment at 48 h (Fig. 8a, b). MMP-9 inhibitor administration exerted a protective effect on both components of ischemic brain injury, namely penumbra and core (Fig. 8e, f). The level of the protective effect of the MMP-9 inhibitor was identical to that of hrIL-1Ra in both core and penumbra lesions of the forebrain (Fig. 8e), meaning that IL-1β and MMP-9 are likely interconnected molecules from the same neuroinflammatory cascade.

### Impact of IL-1Ra on cell death mechanisms

**Fig. 9:**
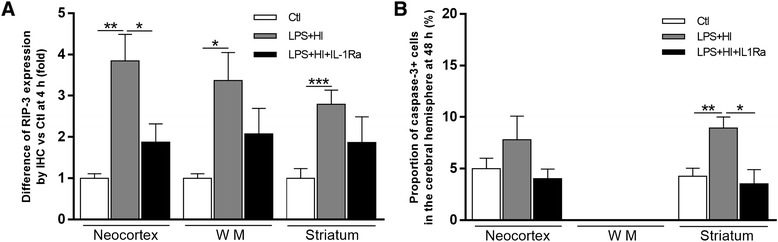
Effect of IL-1Ra on neuronal death effectors in LPS+HI-exposed brains. Decreased relative expression (IHC staining) of RIP-3 within right frontoparietal neocortex, but not in the WM and caudate putamen nuclei exposed to LPS+HI+IL-1Ra compared to LPS+HI at 4 h post-HI (**a**). Following LPS+HI insults, IL-1Ra decreased the percentage of caspase-3+ cells (IHC staining) in the striatum at 48 h post-HI (**b**). *Error bars* represent means ± SEM, four to six animals per conditions, unpaired *t* test with Welch correction, * *p* ≤ 0.05, ** *p* ≤ 0.01, *** *p* ≤ 0.001. Abbreviations: *RIP*-*3* receptor interacting protein 3, *WM* white matter

LPS+HI acted differently on cell death pathway activations depending on forebrain areas. In the LPS+HI-exposed compared to Ctl neocortex, RIP-3-associated necroptosis occurred at 4 h post-HI (Fig. 9a), whereas no change in activated caspase-3 was detected at 4, 24 (data not shown), or 48 h post-HI (Fig. 9a, b). Within the LPS+HI-exposed compared to Ctl striatum, both RIP-3 (at 4 h post-HI) and activated caspase-3 (at 48 h post-HI) were involved in LPS+HI-induced neural cell death (Fig. 9a, b). In the LPS+HI-exposed vs Ctl WM, RIP-3-associated necroptosis was activated at 4 h post-HI (Fig. 9a, b) whereas activated caspase-3 expression was not affected at 4, 24, and 48 h post-HI. IL-1Ra curbed the expression of activated caspase-3 in the LPS+HI-exposed striatum as well as that of RIP-3-associated necroptosis in the LPS+HI-exposed neocortex and WM (Fig. 9a, b).

### IL-1Ra tolerance

**Fig. 10:**
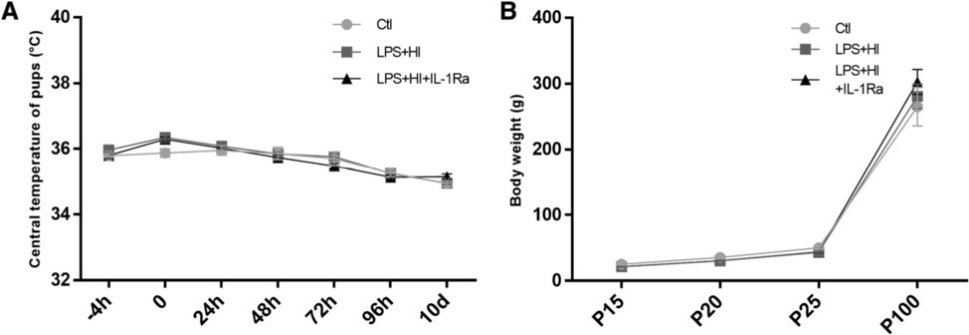
Effect of IL-1Ra on the temperature and the weight growth of LPS+HI-exposed rats. IL-1Ra had no effect on central temperature (**a**) and body weight (**b**) of LPS+HI-exposed rats. *Error bars* represent means ± SEM, eight to sixteen animals per conditions, unpaired *t* test with Welch correction. *Error bars* represent means ± SEM. Abbreviations: °*C* degree Celsius, *g* gram

Follow-up to P120 revealed no mortality following the hypoxic phase in rats exposed to sole LPS+HI vs LPS+HI+IL-1Ra. Beyond those above mentioned, we observed no particular clinical manifestation between animals exposed to LPS+HI vs LPS+HI+IL-1Ra. Since the first injection of IL-1Ra until 10 days later, there was no significant difference in central body temperature between animals from the Ctl vs LPS+HI vs LPS+HI+IL-1Ra experimental groups (Fig. 10a). This means that the neuroprotective and anti-inflammatory effects we observed during—and following—IL-1Ra administration was not due to hypothermia. IL-1Ra did not affect the weight of LPS+HI animals compared to Ctl at P15, P20, P25, and P100 (Fig. 10b).

## Discussion

Our key findings are that IL-1β and MMP-9 from neuronal origin play a central role in LPS+HI-induced injury of the newborn brain. Neuronal IL-1β might be up-regulated both via mRNA synthesis and NLRP1 inflammasome activation. IL-1Ra (peripherally administered) reaches the brain and interacts directly with neuronal cells to interfere with IL-1β mRNA and protein and MMP-9 synthesis. Neuronal IL-1β up-regulates chemokines (CINC-1, MCP-1), as well as other pro-inflammatory and neurotoxic mediators, namely iNOS and MMP-9. MMP-9—likely in combination with iNOS—might participate to disrupt the BBB [23, 24]. BBB leaks, combined to CINC-1 up-regulation, would facilitate PMN infiltration within the brain. IL-1β modulates macrophages’ polarization since its blockade maintains the M1/M2 balance towards an M2 anti-inflammatory and neuroprotective phenotype. Finally, IL-1β activates neural cell death pathways, namely RIP-3-associated necroptosis and caspase-3-mediated apoptosis. Altogether, these results show that IL-1β and MMP-9 contribute to neuronal self-injury and that their blockade curbs this effect.

Pro-IL-1β is cleaved by a macromolecular complex called the inflammasome for IL-1β secretion to occur [25]. Stimuli triggering inflammasome assembly and activation, within LPS+HI-exposed neurons, could be damage-associated molecular patterns (DAMPs) release due to the release of endogenous molecules following cellular damage such as that induced by LPS+HI [20]. Inflammasome-dependent IL-1β processing relies on distinct NLRs that respond to the intracellular milieu and trigger inflammasome assembly [20]. We showed that NLR expression within neural cells is developmentally regulated, NLRP1 being expressed at P12 (but not P1) within neurons. NLRP3 however was undetected in the P1 or P12 immature brain and only expressed later. Thus, exposure to LPS+HI might release DAMPs within the cytoplasm of neurons leading to exacerbated activation of IL-1β, through NLRP1 inflammasome activation. In addition, LPS might interact with TLR-4 to increase the synthesis of IL-1β via NF-κB activation. Such ability of neurons to produce IL-1β mRNA and protein has also been reported by others in rodents subjected to either HI, traumatic or excitotoxic brain injury [26–28]. Thus, exposure to LPS+HI at a specific term-like stage of brain development may lead to an autocrine/paracrine loop of neuronal self-injury, mediated by IL-1β and subsequent neurotoxic mechanisms such as IL-1β-induced MMP-9 and NO production and/or IL-1β exacerbation of excitotoxic damage [25, 29]. Consistent with such mechanisms, IL-1Ra blockade of the IL-1β signaling pathway decreases the extent of LPS+HI-induced brain injury. Another mechanism whereby IL-1Ra limits LPS+HI-induced injury appears to be by preventing the IL-1β-induced M1 polarization of macrophages [30].

MMP-9 competitive inhibitor shrunk the size of LPS+HI-induced brain lesions to a same extent as IL-1Ra treatment. MMP-9 induces cell death via anoikis, an integrin-mediated form of caspase-3-induced apoptosis [31]. When the cell loses its tether to the extracellular matrix, anoikis is induced via caspase-9 and the apoptosome [31]. Such a cell death mechanism is relevant to our findings showing LPS+HI-induction of activated caspase-3 and subsequent neuronal cell death—that is preventable by MMP-9 inhibitor treatment. MMP-9 also alters the BBB by degrading the lamina of intra-cerebral blood vessels. Such disruption would enable pro-inflammatory and/or neurotoxic mediators to leak through the BBB and thus increase the extent of intra-cerebral lesions. NO is also known for its deleterious effects on the BBB [32]. Causing a rise of expression of iNOS in LPS+HI brains, together NO and MMP-9 could alter the BBB permeability.

We also observed cerebral expression of TNFα following LPS+HI exposure (data not shown). It is known that TNFα—the synthesis of which is induced by IL-1β—mediates receptor-interacting-protein (RIP) kinase-dependent necroptosis [33]. RIP-1 and -3 associate with TNF receptor-associated death domain (TRADD) and migrate to the mitochondria [33]. This complex releases reactive oxygen species (ROS) and DNAses from the mitochondria, inducing necroptosis [33]. Accordingly, our findings show that RIP-3 expression is increased after LPS+HI. IL-1β-induced MMP-9 (see Fig. 8) also activates necroptosis via FAS-FAS ligand (FASL) interaction through FAS-associated death domain (FADD) [31, 33]. RIP kinases associated with FADD, as well as TRADD, activate necroptosis [33]. Either of these cell death mechanisms might be involved in LPS+HI-induced brain damage in term NE. In human NE, the only current treatment known to be efficient is hypothermia [34, 35]. Hypothermia acts on penumbra but not core lesions in animal models of neonatal HI encephalopathy [34]. In human NE, hypothermia imparts only partial neuroprotection. IL-1Ra, which acts on both, may one day afford additional neuroprotective benefits to hypothermia [34, 35]. However, this needs to be tested in future clinical trials.

## Conclusion

Interfering with neuronal IL-1β signaling damped neuroinflammation of core and penumbra brain lesions—switch from M1 to M2 macrophage/microglial polarization and decreased PMN infiltration as well as iNOS and chemokine productions—and alleviated motor impairments. Neuroprotective treatments available against NE are very limited. They mainly consist in symptomatic supportive care and in hypothermia that leave about 50 % of the patients affected by moderate or severe neurobehavioral sequelae. rhIL-1Ra, possibly combined to hypothermia, might be a promising translational option to prevent the severe neurodisabilities complicating NE.

## Abbreviations

Ab: antibody
Arg1: arginase 1
BBB: blood–brain barrier
CINC-1: cytokine-induced neutrophil chemoattractant-1
CLAU5: claudin-5
DAMP: danger-associated molecular pattern
DIG: digoxigenin
FADD: Fas-associated death domain
G: gestational day
GFAP: glial fibrillary acidic protein
H&E: hematoxylin eosin
HI: hypoxia/ischemia
hr: human recombinant
Iba-1: ionized calcium-binding adapter molecule 1
ip: intraperitoneal
IF: immunofluorescence
IL: interleukin
IL-1Ra: interleukin-1 receptor antagonist
IHC: immunohistochemistry
iNOS: inducible nitric oxide synthase
LPS: lipopolysaccharide
MCP-1: monocyte chemoattractant protein-1
MMP: matrix metalloprotease
NeuN: neuronal nuclei
NLRP: nod-like receptor protein
P: post-natal day
PAMP: pathogen-associated molecular pattern
PMN: polymorphonuclear neutrophil
Ra: receptor antagonist
RIP: receptor-interacting protein
TNF: tumor necrosis factor
TRADD: TNFα receptor-associated death domain
WM: white matter
